# An unusual complication of Meckel’s diverticulum: Littre’s hernia

**DOI:** 10.11604/pamj.2018.31.243.10740

**Published:** 2018-12-21

**Authors:** Tariq Bouhout, Badr Serji, Ebo Usman Egyir, Benyounes El Amri, Imad Bouhout, Mehdi Soufi, Mohammed Bouziane, Tijani El Harroudi

**Affiliations:** 1 Chirurgie B, CHU Mohammed VI, Oujda, Maroc; 2 Chirurgie A, CHU Mohammed VI, Oujda, Maroc

**Keywords:** Littre´s hernia, Meckel´s diverticulum, intestinal obstruction

## Abstract

Meckel diverticulum is the most common congenital anomaly of the gastrointestinal tract. Any hernia sacs containing Meckel’s diverticulum is called Littre’s hernia. It was described for the first time in 1700 by Alexis Littre. The diagnosis is unlikely to be made preoperatively and surgery is the treatment of choice. We report a rare case of Littre’s hernia who presented with clinical signs of intestinal obstruction.

## Introduction

Meckel’s diverticulum is found at the antimesenteric border of the ileum, usually located from 30 to 90cm from the ileocecal valve. Any hernia containing the Meckel’s diverticulum is termed a Littre’s hernia. Littre’s hernia is a rare anatomoclinical form; it was described for the first time in 1700 by Alexis Littre [1]. The diagnosis is unlikely to be made preoperatively.

## Patient and observation

**Figure 1:**
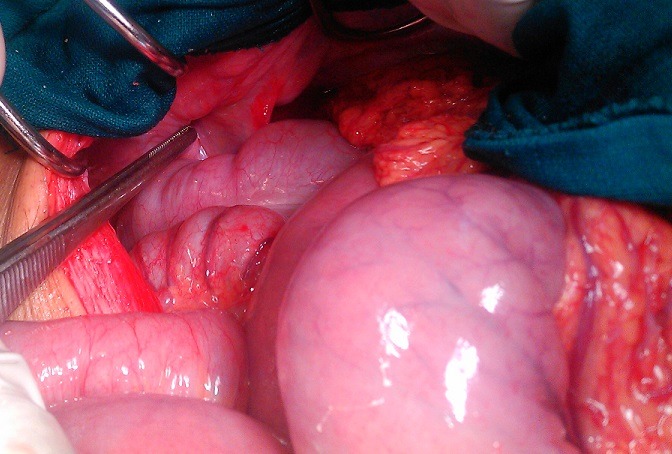
strangulated Meckel’s diverticulum in the sac of obstructed inguinal hernia

**Figure 2:**
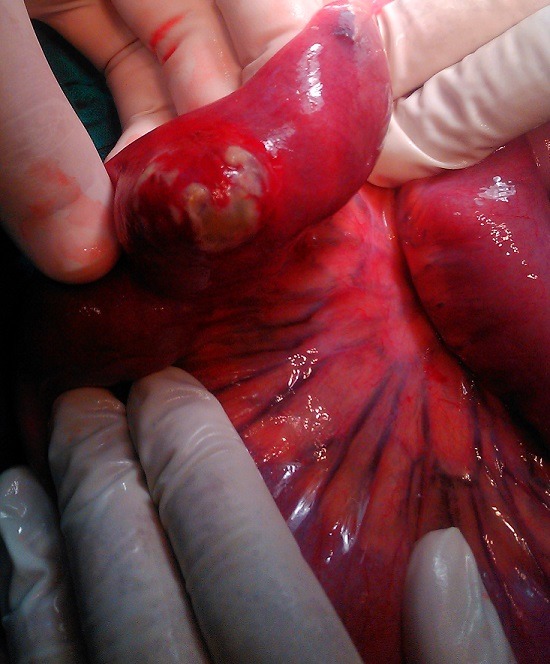
Meckel’s diverticulum located on the antimesenteric surface, 90cm from the ileocecal valve

**Figure 3:**
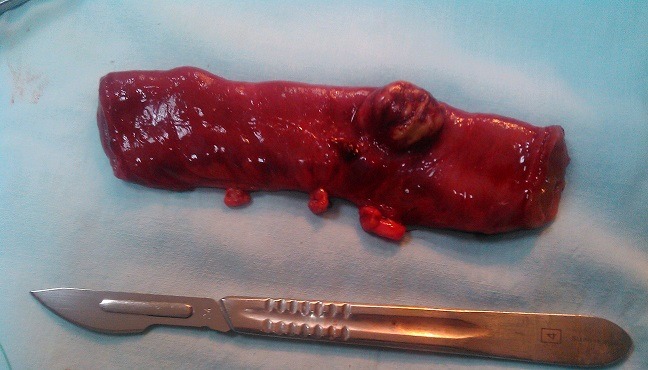
operative specimen

A 39-year-old previously healthy woman was admitted at the emergency service with clinical signs of intestinal obstruction. Physical examination revealed a mass at right inguinal area which was not reducible or pulsatile. Abdominal radiographs showed small bowel obstruction. A diagnosis of strangulated inguinal hernia was made. Operation was done under general anesthesia, a median incision was performed, on exploration a strangulated Meckel’s diverticulum was found in the sac of obstructed inguinal hernia (Figure 1). The diverticulum measured 2cm and was located on the antimesenteric surface, 90cm from the ileocecal valve. Due to the broad base and necrosis of the Meckel’s diverticulum (Figure 2), a small bowel segment was resected (Figure 3) and continuity of ileum restored by an end to end anastomosis. The patient made a rapid and uneventful post-operative recovery.

## Discussion

Meckel’s diverticulum is the most prevalent congenital abnormality of the gastrointestinal tract. The incidence of Meckel’s diverticulum is 2% and normally it is not symptomatic [2]. Meckel diverticulum occur on the antimesenteric border of the ileum, generally at a distance of about 30 to 90cm from the ileocecal valve [3]. In 1700,Alexis Littre, a French surgeon was the first to report three cases of incarcerated femoral hernia containing a small bowel diverticulum [4], since then, hernia sacs containing Meckel’s diverticulum have been called Littre’s hernia [4]. It is a rare anatomoclinical form representing 10% of all complications of Meckel’s diverticulum [1]. Approximately 50% of Littre hernias occur in the inguinal region, 20% in the femoral region, 20% in the umbilical region and 10% in other locations [5]. Because of its low incidence, Littre’s hernia is generally unsuspected and the diagnosis is unlikely to be made preoperatively.

However Sinha reported the first case of an incarcerated Littre hernia diagnosed on computerised tomography [6]. The Meckel diverticulum was seen in the hernia sac as a tubular blind-ending structure arising and communicating with the distal ileum. Surgery is the treatment of choice; the diverticulum is locally excised and small intestine sutured transversely, however if the base is wide and if there is an inflammation at the base of the diverticulum, resection of the involved loop of ileum with anastomosis is the preferred procedure, as was done for our case. So the question arises, it is necessary to resect an incidentally discovered Meckel’s diverticulum? Resection had been the subject of controversy. Some authors advocate the resection of the diverticulum because post-operative morbidity and mortality is very low, whereas others see that resection is not justified because complications are rare. We think that criteria for the resection of Meckel’s diverticulum found incidentally should be suggested.

## Conclusion

An unusual complication of Meckel’s diverticulum, a strangulated Littre’s inguinal hernia is presented. The symptoms and physical findings in this type of hernia are deceptively few; the preoperative diagnosis is usually incarcerated hernia. Surgery is the main stay of treatment.

## Competing interests

The authors declare no competing interests.
